# Mxenes–Au NP Hybrid Plasmonic 2D Microplates in Microfluidics for SERS Detection

**DOI:** 10.3390/bios12070505

**Published:** 2022-07-10

**Authors:** Zhaoxian Chen, Anping Liu, Xiumei Zhang, Jiawei Jiao, Yuan Yuan, Yingzhou Huang, Sheng Yan

**Affiliations:** 1 College of Physics, Chongqing University, Chongqing 400044, China; chenzhaoxian@cqu.edu.cn; 2 Chongqing Key Laboratory of Soft Condensed Matter Physics and Smart Materials, Chongqing University, Chongqing 400044, China; zhangxiumei@cqu.edu.cn (X.Z.); jiaojiawei@cqu.edu.cn (J.J.); 3 State Key Laboratory of Coal Mine Disaster Dynamics and Control, Chongqing University, Chongqing 400044, China; yzhuang@cqu.edu.cn; 4 Institute for Advanced Study, Shenzhen University, Shenzhen 518060, China

**Keywords:** SERS, Mxenes–Au NPs, hybrid plasmonic 2D microplates, microfluidics

## Abstract

Combined with microfluidics, surface-enhanced Raman spectroscopy (SERS) exhibits huge application prospective in sensitive online detection. In current studies, the design and optimization of plasmonic enhanced structures in microfluidics for SERS detection could be an interesting challenge. In this work, hybrid plasmonic 2D microplates composed of Mxenes (Ti_3_C_2_T_x_) microplates and in-situ synthesized Au nanoparticles (Au NPs) are fabricated in a microchannel for enhanced structures in SERS microfluidics. Benefiting from the 2D Mxenes microplates with complex distributions, the enhanced areas generated by Au NPs are quite enlarged in a microchannel, which exhibits high sensitivity in SERS detection at 10^−10^ M for Nile blue (NB) molecules in microfluidics. The mechanism of electromagnetic enhancement (EM) and chemical enhancement (CM) is analyzed. The experimental data indicate the ultrasonic times of Mxenes and the concentration of Au^3+^ play important roles in the sensitivity of SERS detection, which is confirmed by the simulated electric field distributions. Furthermore, a typical pesticide (thiram) at 100 ppm in water is detected on these SERS microfluidics with hybrid plasmonic enhanced structures, which demonstrates that our work not only strengthens the knowledge of plasmonics but also enlarges the application of SERS.

## 1. Introduction

Surface-enhanced Raman spectroscopy (SERS) is a sensing technique, which chiefly originated from the local surface plasmon resonance (LSPR) [1,2,3]. SERS detection is high in sensitivity, stability, and accuracy [4,5,6]. Accordingly, the SERS technique has been covered in substantial arenas for high-precision detection, including human health, material analysis [7,8,9,10,11], environmental pollution [7,12], gas identification [13], food security [14], and biomedicine [15]. Nevertheless, traditional SERS substrates find it difficult to realize real-time detection [16,17], which limits their practical applications. Hence, it is necessary to combine with other technologies. Microfluidics is a miniaturized and automated microsystem with real-time detection [18,19,20]. Many studies report that combining SERS with microfluidics shows extensive applications in sensitive online detection [21,22]. However, the SERS microfluidic chip is of poor molecular enrichment and insufficient space utilization.

To solve these problems, this work proposed hybrid plasmonic 2D microplates composed of Mxenes for SERS-based molecular detection. As a popular series of 2D materials, MXenes have great electron conductivity and an effortlessly manufacturable process, which are widely used in various fields [23]. In addition, there are some literature reports that Ti_3_C_2_T_x_ is used as a SERS substrate. The Ti_3_C_2_T_x_ surface commonly features plentiful and negatively charged electrons, allowing the efficient adsorption and charge transfer between it and the analyte [24,25,26,27]. Mxenes not only have excellent chemical properties, but also have special structure [28,29]. Hence, in this article, the design and optimization of plasmonic enhanced structures in microfluidics for SERS detection could be an interesting challenge [30].

Herein, hybrid plasmonic 2D microplates composed of Mxenes (Ti_3_C_2_T_x_) microplate substrates through in-situ synthesized Au nanoparticles (Au NPs) are fabricated in a microchannel for sensitive SERS detection. Mxenes have three roles in microplate substrates: (1) The decorated MXenes have a large surface area; it is beneficial for target loading, thus, boosting the SERS performance of the Au NPs and suppressing the oxidation of Au NPs. (2) The MXenes can in-situ synthesize Au NPs. (3) Achieve chemical enhancement by MXenes. The limit of detection for this platform can reach up to 10^−10^ M for Nile blue (NB) molecule and 10^−8^ M methylene blue (MB) in microfluidics. Moreover, MXenes transfer electrons directly to the noble metal cations, rendering the formed metallic nanostructures to firmly anchor on MXenes. Moreover, the CM mechanism is explained. Furthermore, thiram at 100 ppm in water is detected on this SERS microfluidics with hybrid plasmonic enhanced structures. Compared with other studies, the hybrid plasmonic 2D microplates suggest the superiorities of high sensitivity, bringing countless possibilities for practical applications in the environment.

## 2. Materials and Methods

### 2.1. Materials

Aluminum titanium carbide (Ti_3_AlC_2_, 99.8%), lithium fluoride (LiF, 98%), hydrochloric acid (HCl, 36–38%), chloroauric acid (HAuCl_4_, 99.99%), polydimethylsiloxane (PDMS, Sylgard184, Dow Corning, Michigan, United States), Nile blue (C_40_H_40_N_6_O_6_S, NB), methylene blue (C_16_H_18_ClN_3_S, MB), and anhydrous ethanol (C_2_H_6_O, 99.7%) were from East Sichuan Chemical Industry, as well as high-purity deionized water (18.2 MΩ cm). All chemicals were used directly, without further purification.

### 2.2. Synthesis of MXenes (Ti_3_C_2_T_x_)

Ti_3_AlC_2_ powder (1 g) and 1 g of LiF were gradually added to 20 mL of HCl at a concentration of 35–38% at 55 °C temperature and reacted for 24 h under magnetic stirring. Then, the Ti_3_C_2_T_x_ solution was obtained by washing the mixture with super pure water to reach pH 6, which sonicates in N_2_ atmosphere for 3 h. Finally, the suspension was washed by centrifugation with deionized water and centrifuged at 1500 rpm for 30 min to obtain multi-layer Ti_3_C_2_T_x_ powder [31].

### 2.3. Preparation of Microfluidics for Mxene–Au NP Hybrid Plasmonic 2D Microplate

Firstly, the MXenes solution was sonicated for 1–5 h to make it evenly dispersed. Then, we used a 1 mL syringe to place 300 μL Ti_3_C_2_T_x_ solution on a microfluidic pump with a flow rate of 5 uL/min. Put the microfluidic pipe on the heated plate with temperature of 60 °C for 30 min. Subsequently, 100 μL different concentrations of HAucl_3_ solution are poached in the microfluidic pump with MXenes (10 uL/min, 1 min). The microfluidic pipe was placed at room temperature for 1 h, and the Au NPs were grown after full reaction with Ti_3_C_2_T_x_ metal acid.

### 2.4. Simulation and SERS Measurement

Computational simulation was conducted using COMSOL Multiphysics. A model that can calculate the three-dimensional electromagnetic field was established. The physical field interface is electromagnetic wave (frequency domain). This work takes steady-state as its object of study and simulates the electric field distribution. At last, the model specific parameters are built using COMSOL Multiphysics (Supplementary Information Table S1). All SERS spectra were recorded using a Raman spectrometer (Horiba IHR550, Horiba Trading Co., Ltd., Shanghai, China.) Microscope (Olympus IX73, Olympus Corporation, Tokyo, Japan). All experimental data were excited with laser of 632.8 nm (MELLES GRIOT 25-LHP-991-230, CVI Melles Griot, Albuquerque, NM, USA), and the integration time was 20 s. SEM images were recorded with field-emission SEM (Mira3 LMH, Tscan).

## 3. Results and Discussion

### 3.1. The Scheme of MXenes–Au NPS Hybrid Plasmonic 2D Microplates in Microfluidics

**Figure 1:**
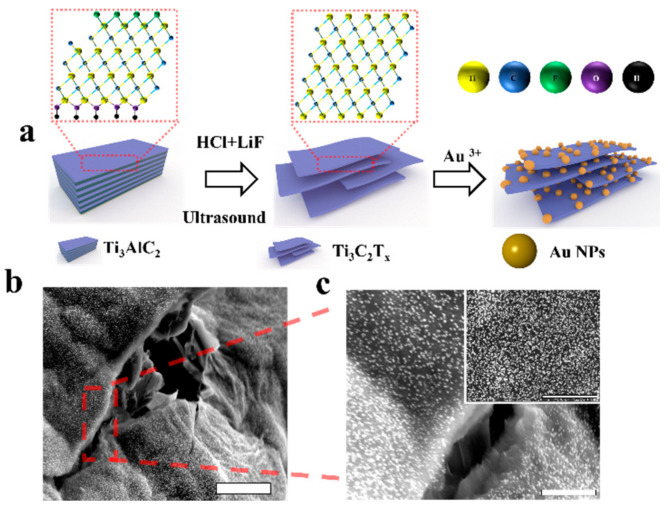
(**a**) Schematic of the hybrid plasmonic 2D microplates composed of Mxenes microplates, the illustration; (**b**) SEM image of the hybrid plasmonic 2D microplates composed of Mxenes microplates, scale bar: 5 μm. (**c**) SEM image was be enlarged, scale bar: 2 μm. The insets are the enlarged SEM image of the hybrid plasmonic 2D microplates with Au nanoparticles at the Mxenes surface, scale bar: 500 nm.

A schematic of the hybrid plasmonic 2D microplates composed of Mxenes microplates is shown in Figure 1a. Ti_3_C_2_T_x_ nanosheets were synthesized by the hydrofluoric acid etching method. In addition, a wrinkle-like structure formed after etching out Al layers from Ti_3_AlC_2_ crystals and in-situ synthesis of Au NPs (Figure 1b,c).

### 3.2. Effect of the Thickness of MXenes on SERS Sensitivity

**Figure 2:**
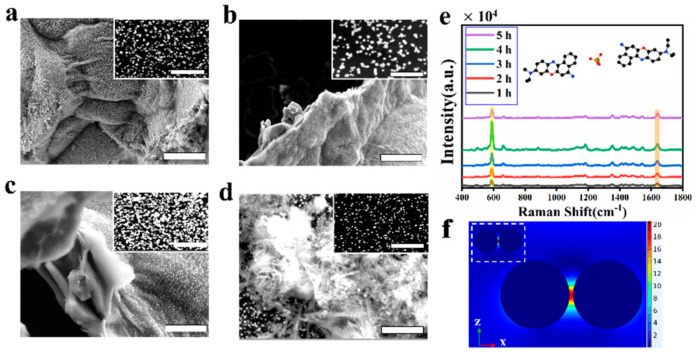
Structural characterization of MXenes–Au NP hybrid plasmonic 2D microplates: (**a**–**d**) SEM image of MXenes–Au NPs 2D microplates; (**a**) Mxenes are ultrasonicated for 1 h, (**b**) 3 h, (**c**) 4 h, and (**d**) 5 h. Scale bar: 5 μm, the illustration was enlarged 2.5-times, scale bar: 2 μm. (**e**) SERS spectra of NB at a concentration of 1 × 10^−6^ M acquired from the different hours. (**f**) The electromagnetic field of Au NPs with a gap of 10 nm. The inset shows Au NPs with a gap of 5 nm.

SERS signal intensity is related to the molecular concentration, nanoparticle size, and “hot spot” density. Since the MXene substrate decorated with Au NPs has many layers, this will generate numerous hot spots. Therefore, in this work, the SERS signal strength is related to different thickness of Mxenes. The MXenes connect with the -OH bond, such as Ti_3_C_2_(OH)_2_ and Ti_3_C_2_O(OH). When -OH bond of MXenes contacts with the acid solution, the electrons will be transferred to the chlorotic acid solution and provide electrons to the precious metal cation. The Au^3+^ is reduced to Au nanoparticles [32]. The increasing Ti oxidation state may lead to material structural reconstruction. MXenes has a non-bonded Ti bond, which provides a frequency band with many electrons, thus, rendering structural stability of MXenes. In the theory, as an electron acceptor, Au^3+^ can gain electrons, being low-density Au, while MXenes as an electron donor should be oxidized. To sum up, this work needs to study the effect of the thickness of MXenes on SERS sensitivity. The original MXenes solution is placed in ultrasonic machine and then different times are regulated to obtain the different thicknesses of MXenes nanosheets. Figure 2a shows the Au NPs are inhomogeneously distributed on the surface of 2D MXenes nanosheets, when Mxenes were ultrasonicated for 1 h. The Au NPs have different distance. The MXenes with larger thickness have larger specific surface area, shown in Figure S1a. When the Mxenes were ultrasonicated for 2 h and the Au NPs are distributed on the surface of 2D MXenes nanosheets (Figure 2b), the thickness of MXenes nanosheets decreases (Figure S1b). Figure 2c shows that the Mxenes are ultrasonicated for 4 h, which are divided into many layers, and each layer has a varying degree of folding and bending (Figure S1c). The Au NPs are homogeneously distributed on the surface of 2D MXenes nanosheets, increasing the interparticle hotspot aggregation and improving the SERS sensitivity. Figure 2d expresses that the SEM image of MXenes–Au NP hybrid plasmonic 2D microplates, when the Mxenes are ultrasonicated for 5 h. The thickness of MXenes nanosheets is very sparse (Figure S1d). The Au NPs are dispersedly distributed on the surface of 2D MXenes nanosheets. Because MXenes are excessively ultrasonicated, the large area structures of MXenes are destroyed. The smaller specific surface area of MXenes provided fewer adsorption sites. Therefore, the Raman probe molecules are hardly adsorbed. Besides, Figure 2e shows that MXenes thickness is related to SERS spectra. The NB molecule is selected as the Raman probe molecule. The concentration of the NB molecule is 1.0 × 10^−6^ M. The Au NPs were grown after full reaction with Ti_3_C_2_T_x_ metal acid in a microfluid channel. As such, 300 μL NB molecule solution was pumped into the channel with a flow rate of 10 μL/min. The inhibition time for the samples lasted for 30 min. Then, a laser wavelength of 632.8 nm irradiated the hybrid plasmonic 2D microplates with an integration time of 20 s. Figure 2e shows that the strongest SERS intensities are at 587 ${\mathrm{cm}}^{-1}$ when the Mxenes are ultrasonicated for 4 h. According to the SEM image of the MXenes–Au nanoparticles (Figure 2a–d), the average diameter of Au NPs is about 250 ± 10 nm, but the particle spacing varies. To analyze the enhancement mechanism, the finite element method (FEM) commercial package (COMSOL) was used to simulate the electric field distribution of neighboring Au nanoparticles, shown in Figure 2f. The photograph reveals the electromagnetic field distribution simulation of Au NPs with different distances (10 nm and 5 nm). The light is propagating along the -z direction (normal to film surface, green arrow) with polarization along the x direction (parallel to dimer, red arrow). The results show that the closer spacing between Au NPs brings stronger electromagnetic fields and, thus, the Raman signal will be enhanced. When the particle spacing decreases, the plasmonic structure of the particle resonance wavelength shifts. The consequence is the different spacing between Au NPs will affect the distribution of electromagnetic field, so the Raman signal will be enhanced to varying degrees. When Mxenes were ultrasonicated for 4 h, the maximum of SERS intensity was obtained. A few layers of MXenes were spread out in the microchannel, and the sheet was just a nanometer-thin blanket, providing a basis for SERS signal collections with the high homogeneity. The space utilization of the microfluidic pipeline was greatly optimized.

### 3.3. Effect of the Concentration of Au^3+^ on SERS Sensitivity

**Figure 3:**
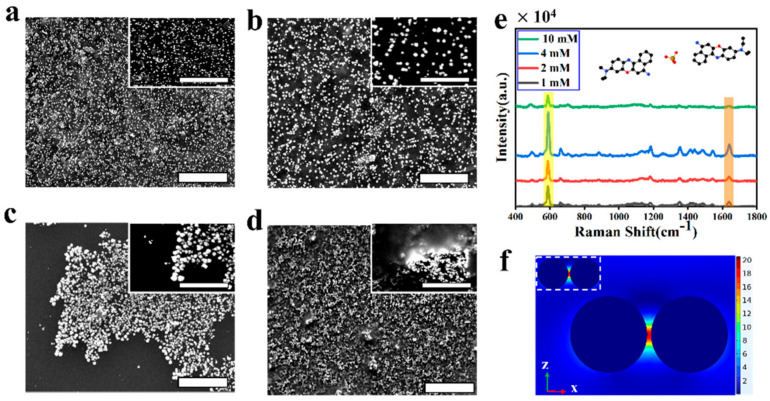
Structural characterization of MXenes–Au NP hybrid plasmonic 2D microplates: (**a**–**d**) SEM image of MXenes–Au NP 2D microplates; the concentrations of HAuCl_4_ (**a**) 1 mM, (**b**) 2 mM, (**c**) 4 mM, and (**d**) 10 mM. Scale bar:5 μm. The illustration was enlarged 2.5-times, scale bar: 2 μm. (**e**) SERS spectra of NB at a concentration of 1 × 10^−6^ M acquired from the different concentrations of HAuCl_4_. (**f**) The electromagnetic field of Au NPs with sizes of 200 nm. The inset shows the Au NPs with a size of 100 nm.

The SERS signal strength is related to the different concentration of Au^3+^, with HAuCl_4_ concentrations of 10 mM, 4 mM, 2 mM, and 1 mM. Figure 3a displays the SEM image of the hybrid plasmonic 2D microplates composed of Mxenes microplates with Au NPs, when the concentration of HAuCl_4_ is 1 mM. The Au NPs have a uniform morphology with an average particle size of 140 nm, and all the Au NPs are limited within the MXenes sheets. Figure 3b shows the concentration of HAuCl_4_ with 2 mM, where the size of Au NPs becomes larger. The SEM image of the MXenes–Au NP hybrid plasmonic 2D microplates with the concentrations of HAuCl_4_ (4 mM) is shown in Figure 3c. Obviously, the Au NPs are distributed on the MXenes evenly and the spacing of nanoparticles is very concentrated. The region of hot spots becomes more uniform to improve the SERS sensitivity. Figure 3d shows concentrations of HAuCl_4_ of 10 mM. With the high concentration of Au^3+^, the MXenes provide less electrons with Au^3+^. Hence, The SERS signal enhancement is greatly weakened. Finally, Figure 3e shows that the concentrations of HAuCl_4_ are dependent on the SERS spectra of the NB molecule at a concentration of 1.0 × 10^−6^ M taken with 632.8 nm lasers. According to the SEM images of the MXenes–Au nanoparticles (Figure 3a–c), the average diameter of Au NPs is about 100 to 250 nm. To further analyze the enhancement mechanism, the finite element method (FEM) commercial package (COMSOL) was used to simulate the electric field distribution of two Au nanoparticles, shown in Figure 3f. When the particle size increases, a shift in the plasmon absorption band was observed. Figure 3f shows the electromagnetic field of Au NPs with size (100 nm and 200 nm). The SERS intensity is maximum when the best concentrations of HAuCl_4_ are 4 mM.

### 3.4. SERS Activity of the Mxenes–Au NP Hybrid Plasmonic 2D Microplate Substrate

**Figure 4:**
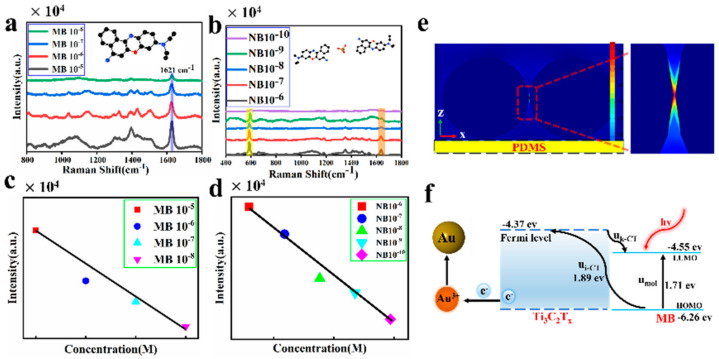
SERS performance of the hybrid plasmonic 2D microplates composed of MXenes–Au NP microplates. Excitation is a 632.8 nm laser. The acquisition time of all SERS spectra is 20 s. SERS spectra of (**a**) MB, (**b**) NB for different concentrations acquired from the hybrid plasmonic 2D microplates composed of MXenes–Au NP microplates (**c**) SERS intensity at 1621 cm^−6^ as a function of MB concentrations (**d**) SERS intensity at 587 cm^−1^ as a function of NB concentrations. (**e**) Electric field distribution of 100 nm Au nanoparticle on PDMS surface. (**f**) Mechanism of CM.

The SERS sensitivity of the MXenes–Au NP hybrid plasmonic 2D microplate substrates is investigated. The MB and NB are two illustrative organic Raman molecules. Figure 4a demonstrates the SERS spectra of NB have various concentrations (1.0 × 10^−5^, 1.0 × 10^−6^, 1.0 × 10^−7^, and 1.0 × 10^−8^ M); they were collected from the SERS microfluidics substrate. The robust characteristic Raman band at 1621 ${\mathrm{cm}}^{-1}$ can be distribution to the C−C bond. The strength of the characteristic peak reduces with the decreasing MB concentrations. When the MB concentration is decreased to 10^−8^ M, the characteristic peak can be obviously separated. The SERS measurements were performed using a 632.8 nm laser with an acquisition time of 20 s for each spot. Figure 4c demonstrates that the SERS intensities at 1621 ${\mathrm{cm}}^{-1}$ are related to the MB concentrations. Clearly, the relationship between the peak intensity and the concentrations exhibits a good linearity. In addition, the NB concentrations (1.0 × 10^−6^, 1.0 × 10^−7^, 1.0 × 10^−8^, 1.0 × 10^−9^, 1.0 × 10^−10^ M) decreased and the SERS intensity decreased with the characteristic peaks at 587 cm^−1^ and 1630 cm^−1^ (Figure 4b). Figure 4d shows the SERS intensities at 587 ${\mathrm{cm}}^{-1}$ are related to the NB molecule concentrations. Figure 4e shows the electric field of 100 nm Au nanoparticle distributed on PDMS surface. The SERS enhancement mechanisms originate from EM and CM. The local electromagnetic field is enhanced by the Au NPs. On the surface of MXenes, the electrons will be transferred. Figure 4f shows the mechanism of CM. Firstly, the MB molecule produces the molecular transition ($\mu_{mol}$), from the highest molecular orbital (HOMO) to the lowest unoccupied molecular orbital LUMO, the Ti_3_C_2_T_x_ fermi level. When the energy of the laser is consistent with the energy transition of the MB molecule, the laser will trigger the molecular transfer resonance and then enhance the SERS signal. The charge transfer transition ($\mu_{i-CT}$) is from the molecular HOMO to the fermi level of Ti_3_C_2_T_X_. The transition ($\mu_{k-CT}$) is from the fermi level of Ti_3_C_2_T_X_ to the molecular LUMO [32,33,34]. The SERS signal enhancement is related to laser wavelength. Lombardi and Birke [35,36] put forward a unified theory in Formula (1).

(1) $$R_{iKF}=\frac{\left[\mu_{i-CT}\mu_{mol}\mu_{k-C\;T}i|Q_{k}|k\right]}{\left[\left({\left(\varepsilon_{1}\left(\omega \right)+2\varepsilon_{0}\right)}^{2}+\varepsilon_{2}{}^{2}\right)\left(\omega_{i-CT}{}^{2}+\omega^{2}+\gamma_{i-CT}{}^{2}\right)\left(\omega_{mol}{}^{2}+\omega^{2\;}+\gamma_{mol}{}^{2}\right)\right]}$$

The real and imaginary parts of the permittivity of the SERS substrate are $\varepsilon_{1}$ and $\varepsilon_{2}$, and $\varepsilon_{0}$ is the real part of the permittivity of solution. ω is the laser frequency ($4.5^{10}\;\mathrm{HZ}$), $\omega_{i-CT}\;\mathrm{and}\;\omega_{mol}$ are the frequency associated with the respective processes; $\gamma_{i-CT}$ is the damping factors of charge-transfer, and $\gamma_{mol}$ is the damping factors of molecular transition. The calculation result shows the 632.8 nm laser energy is 1.96 ev, close to the energies of charge transfer and molecular transitions [37]. Therefore, the hybrid plasmonic 2D microplates composed of MXenes–Au microplate substrates will be more effective than MXenes alone. In order to evaluate the stability of the SERS substrate, we randomly selected nine points on MXenes–Au microplates. Figure S2 shows the changes in SERS intensity of Raman molecule (NB) from the MXenes–Au microplates, indicating that the stability of the SERS substrate was improved with an acceptable RSD of 16.35%. In addition, we also investigated the reproducibility of the SERS substrate. Figure S3 shows the Raman spectral signals collected on the same substrate for seven days. Those experimental data show that the SERS substrate has excellent reproducibility.

### 3.5. Detection of Thiram

**Figure 5:**
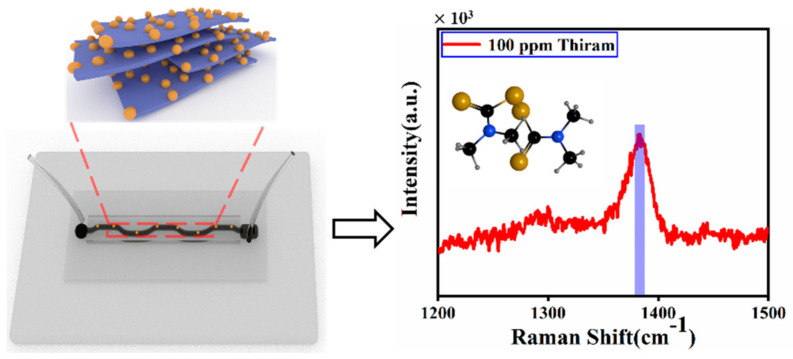
Schematic of the SERS detection in solution.

As a SERS nano sensor, the feasibility of the hybrid plasmonic 2D microplates is further evaluated using organic pesticide pollutants. The poisonousness of thiram at low concentrations has become a concern for environmental safety. There are many SERS methods that have been reported to detect thiram. The bimetallic core shelled nanoparticles were synthesized for rapid detection of thiram in solution milk using SERS, the thiram with limit of detection (LOD) of 0.21 ppm [38]. In addition, they also reported two-dimensional self-assembled Au–Ag core-shell nanorods nanoarray and detected the thiram with LOD of 18 ppm [37]. However, those methods cannot detect thiram in a flowing solution environment. SERS detection is performed simultaneously with the injection of thiram solutions into the microfluidics channel. The thiram was pumped into the channel at a flow rate of 10 μL/min for 10 min. Figure 5 shows the Raman spectra for thiram. The sturdiest Raman characteristic peak of 1380 cm^−1^ is attributed to the C–N vibrating. In a word, thiram at 100 ppm in water is detected on the SERS microfluidics with hybrid plasmonic enhanced structures, demonstrating that this platform can not only strengthen the knowledge of plasmonics, but also enlarge the application of SERS.

## 4. Conclusions

The combination of microfluidic SERS substrates and Ti_3_C_2_T_x_ MXenes was proposed for solution detection. The MXenes–Au NP hybrid plasmonic 2D microplate substrates can simultaneously produce CM and EM, which shows an extraordinary sensing performance, including high sensitivity. The SERS function of substrates is tested using NB and MB. The experimental dates indicate the ultrasonic times of Mxenes and the concentrations of Au^3+^ play important roles in the sensitivity of SERS detection. Benefiting from the 2D Mxenes microplates with complex distributions, the enhanced areas generated by Au NPs are quite enlarged in the microchannel, which enables high sensitivity for SERS detection. The limit of detection can reach as low as 10^−10^ M for the NB molecule and 10 ^−8^ M for the MB molecule in the microfluidic channel. This hybrid plasmonic 2D microplate substrate was demonstrated to detect thiram, showing a great capability for environment monitoring.

## Supplementary Materials

The following supporting information can be downloaded at: https://www.mdpi.com/article/10.3390/bios12070505/s1, Figure S1. SEM micrographs of Mxenes (a) Mxenes ultrasonicated for 1 h. (b) Mxenes ultrasonicated for 3 h. (c) Mxenes ultrasonicated for 4 h. (d) Mxenes ultrasonicated for 5 h. Figure S2. (a) Raman spectra of 9 random detection points on Mxenes–Au microplates. (b) SERS intensity of NB at 587 cm^−1^ from 9 random detection points. The concentration of NB is 1 × 10^−6^ M. Figure S3. (a) Raman spectra of NB at a concentration of 1 × 10^−6^ M from the seven days (b) SERS intensity of NB at 587 cm^−1^ from different days. Table S1. specific parameters.
