# Supplementary material for: Mxenes–Au NP Hybrid Plasmonic 2D Microplates in Microfluidics for SERS Detection
Source: Biosensors (Basel). 2022 Jul 10;12(7):505. doi: 10.3390/bios12070505 (PMC9312844; doi:10.3390/bios12070505)
Supplement: Supplementary file 1 [file biosensors-12-00505-s001.zip › biosensors-1756840-supplementary.pdf]

# Supporting information

# for

# Mxenes-Au NPs Hybrid Plasmonic 2D Microplates in Microfluidics for SERS Detection

**Zhaoxian Chen [1], Anping Liu [1,*], Xiumei Zhang [2], Jiawei Jiao [2], Yuan Yuan [2,*], Yingzhou Huang [3], Sheng Yan [4,*]**

1. College of Physics, Chongqing University, Chongqing 400044, China
2. Chongqing Key Laboratory of Soft Condensed Matter Physics and Smart Materials, Chongqing University, Chongqing 400044, China
3. State Key Laboratory of Coal Mine Disaster Dynamics and Control, Chongqing University, Chongqing 400044, China
4. Institute for Advanced Study, Shenzhen University, Shenzhen 518060, China

* Correspondence: liuanping@cqu.edu.cn (A.L.); yuanyuan9215@gmail.com (Y.Y.); shengyan@szu.edu.cn (S.Y.);

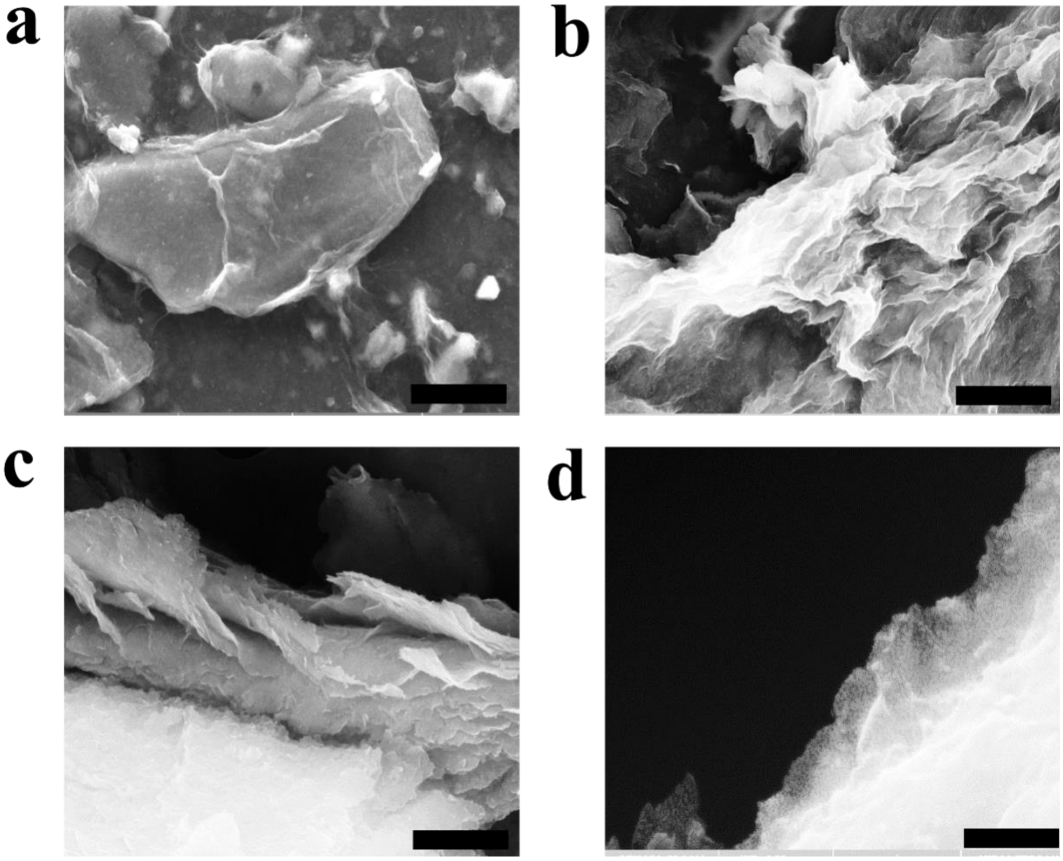


**Figure S1.** SEM image of Mxenes. (a) Mxenes are ultrasonicated for 1 h. (b) 3 h. (c) 4 h. (d) 5 h. Scale bar: 5 μm（a and b）and 2 μm (c and d).

**Table S1**. specific parameters.

| Name | Expression | Unit | Value |
|---|---|---|---|
| F | $c\_const/\omega l$ | | $4.7361 \times 10^{14}\ 1/s$ |
| ωl | 633 | [nm] | $6.33 \times 10^{-7}$ m |
| sigma_geom | $pi \times radius^2$ | | $7.854 \times 10^{-15}\ m^2$ |
| S_in | $E0^2/2 \times Z0\_const$ | | $0.0013272\ W/m^2$ |
| radius | 100 | | $5 \times 10^{-8}$ m |
| E0 | 1 | [nm] | $1\ V/m$ |

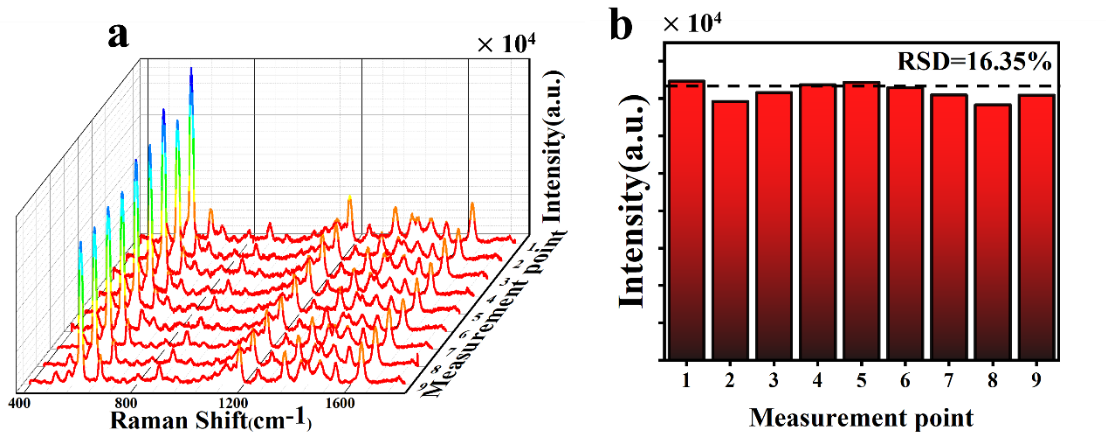


Figure S2. (a) Raman spectra of 9 random detection points on Mxenes-Au microplates. (b) SERS intensity of NB at 587 cm$^{-1}$ from 9 random detection points. The concentration of NB is $1 \times 10^{-6}$ M.

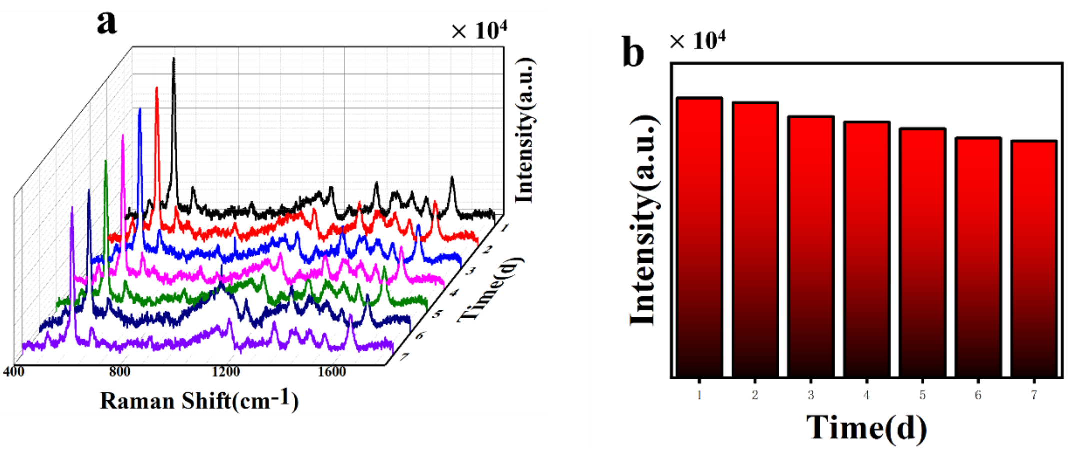


Figure S3. (a) Raman spectra of NB at a concentration of $1 \times 10^{-6}$ M from the seven days (b) SERS intensity of NB at 587 cm$^{-1}$ from different days.
